# Shigellosis: An Old Disease in New Clothes?

**DOI:** 10.1371/journal.pmed.0030354

**Published:** 2006-09-12

**Authors:** Philippe J Sansonetti

## Abstract

Sansonetti discusses a "landmark" study of the epidemiology of shigellosis, involving over 600,000 people of all ages in six Asian countries.

A new study published in *PLoS Medicine* by von Seidlein and colleagues is a landmark in research on shigellosis [1]. von Seidlein and colleagues conducted a prospective, population-based, multi-centre study of *Shigella* diarrhoea in six Asian countries, producing data on disease burden, clinical manifestations, and microbiology. The study provides, at the scale of a continent, critical epidemiological information regarding prevention strategies against a largely neglected disease that threatens to re-emerge due to its highly infectious potential and its ability to develop resistance to multiple drugs [2].

## The Burden of Diarrhoeal Disease

There is no sign that the incidence of diarrhoeal diseases, which are diseases of the poorest, is currently decreasing. Morbidity remains high, particularly in children younger than the age of five years (3.2 diarrhoea episodes per child per year), according to active surveillance studies carried out between 1992 and 2000 [3]. With 2.5 million annual deaths, diarrhoea remains a leading public health concern, even though the attributed mortality has steadily declined in endemic areas, from 13.6 children per 1,000 per year in the period 1954–1979 to 4.9 children per 1,000 per year in the period 1992–2000 [3]. In other words, mortality has roughly halved in the past 20 years. Better primary care, education of mothers, and widespread implementation of oral rehydration therapy are probably the factors that significantly contributed to the decreasing trends in mortality.

Evaluating the health impact of diarrhoea and implementing therapeutic and preventive measures is a headache for national and international health authorities. Unlike other infectious diseases, such as tuberculosis, HIV/AIDS, and malaria, which are all caused by a single etiological agent, diarrhoea is etiologically diverse. Setting priorities for tackling diarrhoeal disease is a complex endeavour, and this complexity largely accounts for its status as a neglected disease. Priorities nonetheless emerge, such as the need to tackle cholera and cholera-like infections caused by enterotoxigenic *Escherichia coli*, rotavirus infection, and shigellosis. Another priority is vaccinedevelopment. Rotavirus vaccines are currently appearing on the market, and promising vaccines against the other pathogens that cause diarrhoea are in the pipeline [4].

The time has come to update epidemiological and disease burden data on diarrhoeal diseases.

The time, therefore, has come to update epidemiological and disease burden data on diarrhoeal diseases, particularly for shigellosis. In a retrospective, literature-based study on shigellosis published in 1999 [5] that has become the “gold standard”, projections suggested a mean annual morbidity of 165 million cases and a mean annual mortality of 1.1 million cases, 69 percent occurring in children younger than five years. But these projections were based upon retrospective studies, and so there is clearly a need to revisit shigellosis epidemiology to see if it is following the global trend of diarrhoeal diseases, or if it has its own unique evolutionary profile.

More recent epidemiological studies carried out in endemic areas raise questions about the actual burden of the disease and certainly challenge the widely accepted idea of a decline in the incidence of shigellosis. For instance, a recent population-based study conducted in Thailand during a period of 36 months showed that estimates of incidence are 10- to 100-fold higher than those found by routine, health ministry–based surveillance [6]. Such studies confirm that shigellosis is generally largely underestimated.

There are three major reasons for this underestimation. First, published studies are often based upon passive surveillance carried out in medical institutions, and, therefore, many cases are missed, particularly those devoid of dysenteric symptoms that do not justify medical attention. Whether what counts in public health decisions are exhaustive figures on the incidence across the entire population or simply data on the cases that come to medical attention and bear medical costs is, of course, an important issue. Nevertheless, accurate appreciation of circulating strains in the population is important for decision makers, particularly for a disease that has no significant reservoir other than humans, making it a good target for eradication by a vaccine.

Second, *Shigella* is a very fastidious microorganism that “dislikes” transport, and for which no enrichment medium exists [7]. There is, therefore, a clear need for improved, quick, and robust methods of straight detection from faecal samples. Last but not least, significant numbers of cases occur in adults, with a second disease peak after the age of 40 years. These cases are not necessarily considered in studies that are often focused on young children.

One major issue is at stake here: do we still need a vaccine against shigellosis [8]? If the morbidity and mortality from shigellosis turn out to be declining, one could seriously question the relevance of continued efforts towards developing a vaccine. Such efforts have not, so far, attracted significant interest from vaccine companies, and the idea of developing such a vaccine may become even less appealing if its cost–benefit ratio were to further decline. On the other hand, if the burden of shigellosis is *not* falling, epidemiological studies would be crucial in deciding which type of vaccine should be developed, particularly with respect to the number of serotypic valences represented (protection against *Shigella* infection is generally considered serotype specific).

## A Landmark Study

von Seidlein and colleagues' study of the epidemiology and microbiology of shigellosis was conducted in study sites in three rural or semi-rural areas (China, Vietnam, and Thailand) and three urban slums (Bangladesh, Pakistan, and Indonesia). The authors used passive surveillance for case detection, which depends on the health care–seeking behaviour of individual patients, and which risks missing a significant number of cases. Nevertheless, the authors included more than 600,000 people of all ages, which is an impressive achievement. Five major points emerged from their study.

First, the researchers found that, overall, the incidence of diarrhoeal diseases remains high (40 per 1,000 per year in all age groups, and as high as 254 per 1,000 per year in children younger than five years). *Shigella* infection, diagnosed by classical microbiology procedures, accounted for five percent of these diarrhoeal episodes, a proportion very similar to that found in previous studies [5].

Second, the researchers also used real-time polymerase chain reaction (PCR), which can amplify the gene coding for the invasion plasmid antigen H *(ipaH)*, and which is a more sensitive diagnostic technique than classical methods. PCR detected *ipaH* in 33 percent of a sample of culture-negative stool specimens, suggesting that classical methods may miss a lot of cases. PCR itself has not yet become globally accepted, due to the potential for false positives and difficulties of implementation in the field. von Seidlein and colleagues' study emphasizes the urgent need to develop robust, sensitive, and specific *Shigella* diagnostic tools that could detect isolates straight from the faecal material. Promising results have recently been obtained with immunochromatographic techniques (that is, dipstick; F. Nato, A. Phalipon, P. L. Nguyen Thi, P. Sansonetti, Y. Germani, unpublished data).

Third, in von Seidlein and colleagues' study, mortality associated with shigellosis was lower than that found in previous epidemiological studies, and the current work did not demonstrate high numbers of late complications in the 90 days of follow-up. This trend towards a more benign pattern of shigellosis needs explanation. The better nutritional status of children in economically emerging Asian countries is a likely possibility, given the significant impact of malnutrition on mortality [9]. Other possible explanations are quicker access to and improvement of primary care, and more extensive use of antibiotics. A global change in the diverse *Shigella* serotypes is a highly unlikely explanation. *Shigella dysenteriae* type 1, the Shiga bacillus, which has the highest attack rate and which is most commonly responsible for severe cases (due to expression of the Shiga toxin) is virtually absent as an epidemic strain at the moment in Asia. This absence was confirmed by the current study.

Fourth, a major contribution of this study is the demonstration of the high diversity of serotypes that are significantly represented overall. With the exception of Thailand, in which, as is already known, *S. sonnei* prevails, in other areas the authors found a large diversity of serotypes. A serogroup such as *S. boydii* that was considered rare and limited to the Indian subcontinent has now expanded its “zone of activity”. This makes the aim of developing a serotype-based *Shigella* vaccine a difficult endeavour, and emphasises the need for a switch in paradigm and investment in renewed research efforts to develop cross-protective vaccine candidates.

A last, but essential, point is the demonstration of the high incidence of multiple drug resistance, including all first-line antibiotics (chloramphenicol, tetracycline, sulfonamides, ampicillin, sulfamethoxazole-trimethoprim, and nalidixic acid). This is not a new finding, but what is important is the demonstration that in some areas a significant percentage of strains (about five percent) is also resistant to fluoroquinolones, leaving little, if any, therapeutic alternative. Large, uncontrolled use of antibiotics in these areas is likely to provoke a major crisis, which may change the profile of shigellosis once again, but for the worse.

## Conclusion

von Seidlein and colleagues found that the incidence of shigellosis is unchanged, but that there has been a trend towards a more benign disease. Is this sufficient to reconsider the relevance of developing a *Shigella* vaccine? The answer is likely to be no. We still do not know the reasons why the disease has become less deadly, and it is possible that the disease could become more deadly in the future, especially with the rapid spread of multi-drug resistance and the return of *S. dysenteriae* type 1. Three-quarters of a century ago, Charles Nicolle had already warned us against the *“génie évolutif”* of infectious diseases. Remember also that a “giant” is absent from this large-scale study and from others: Africa.

## Abbreviations

*ipaH*: invasion plasmid antigen H
PCR: polymerase chain reaction
